# Interleukin-1β Inhibition for Chronic Kidney Disease in Obese Mice With Type 2 Diabetes

**DOI:** 10.3389/fimmu.2019.01223

**Published:** 2019-05-29

**Authors:** Yutian Lei, Satish K. Devarapu, Manga Motrapu, Clemens D. Cohen, Maja T. Lindenmeyer, Solange Moll, Santhosh V. Kumar, Hans-Joachim Anders

**Affiliations:** ^1^Medizinische Klinik and Poliklinik IV, Klinikum der Universität München, Munich, Germany; ^2^Division of Nephrology, Krankenhaus Harlaching, Munich, Germany; ^3^Institute of Clinical Pathology, University Hospital Geneva, Geneva, Switzerland

**Keywords:** chronic kidney disease, fibrosis, NLRP3 inflammasome, uremia, diabetes

## Abstract

Inflammasome-driven release of interleukin(IL)-1β is a central element of many forms of sterile inflammation and has been evident to promote the onset and progression of diabetic kidney disease. We microdissected glomerular and tubulointerstitial samples from kidney biopsies of patients with diabetic kidney disease and found expression of IL-1β mRNA. Immunostaining of such kidney biopsies across a broad spectrum of diabetic kidney disease stages revealed IL-1β positivity in a small subset of infiltrating immune cell. Thus, we speculated on a potential of IL-1β as a therapeutic target and neutralizing the biological effects of murine IL-1β with a novel monoclonal antibody in uninephrectomized diabetic db/db mice with progressive type 2 diabetes- and obesity-related single nephron hyperfiltration, podocyte loss, proteinuria, and progressive decline of total glomerular filtration rate (GFR). At 18 weeks albuminuric mice were randomized to intraperitoneal injections with either anti-IL-1β or control IgG once weekly for 8 weeks. During this period, anti-IL-1β IgG had no effect on food or fluid intake, body weight, and fasting glucose levels. At week 26, anti-IL-1β IgG had reduced renal mRNA expression of kidney injury markers (Ngal) and fibrosis (Col1, a-Sma), significantly attenuated the progressive decline of GFR in hyperfiltrating diabetic mice, and preserved podocyte number without affecting albuminuria or indicators of single nephron hyperfiltration. No adverse effect were observed. Thus, IL-1β contributes to the progression of chronic kidney disease in type 2 diabetes and might therefore be a valuable therapeutic target, potentially in combination with drugs with different mechanisms-of-action such as RAS and SGLT2 inhibitors.

## Introduction

Type 2 diabetic mellitus (T2DM) is systemic disorder and global health concern. Chronic kidney disease (CKD) is common in T2DM and largely contributes to morbidity and mortality. Diabetic and non-diabetic nephropathies or a combination of both contribute to the progression of CKD in T2DM and all involve a contribution of local and systemic sterile inflammation driving kidney atrophy (1). Sterile inflammation is a consequence of danger signals released from cells under glycotoxic, oxidative, or other forms of stress activating inflammasomes and other pattern recognition receptors (2, 3). For example, the NLRP3 inflammasome induces the assembly of several cytosolic proteins ultimately leading to activation of caspase-1, which promotes the enzymatic activation and secretion of mature IL-1β (4, 5). IL-1β activates ubiquitously expressed IL-1 receptors inducing numerous pro-inflammatory mediators (6). Given its central role in orchestrating sterile inflammation, IL-1, or IL-1R-targeting therapeutics have been proven effective in hereditary fever syndromes, Still disease, juvenile arthritis, gout, and cardiovascular events in including patients with T2DM (7–12). The evidence supporting the mechanistic concept that IL-1β would drive systemic inflammation and vascular injury in diabetes is less consistent (10, 13–17).

Two preclinical studies support targeting the NLRP3-IL-1-IL-1R axis in T2DM-related CKD. Shahzad et al. found *db/db* mice with T2DM to be protected from kidney disease by injecting the human recombinant IL-1R antagonist anakinra (18). Orellana et al. found that anti-IL-1β IgG reduced urinary TNF-α levels in T1 diabetic DBA/2J mice (19).

We therefore speculated that a IL-1β-neutralizing antibody could have protective effects on CKD progression in T2DM. To address this concept, we performed an interventional study using uninephrectomized obese *db/db* mice with T2DM and CKD, a model previously validated to predict the outcome of clinical trials on diabetic kidney disease (20–23).

## Materials and Methods

### Human Kidney Biopsy Transcriptomics

Human renal biopsies from patients with diabetic nephropathy (DN) (*n* = 7) and livinv donor (LD) controls (*n* = 18) were collected within the framework of the European Renal cDNA Bank—Kröner-Fresenius Biopsy Bank (24). Biopsies were obtained from patients after informed consent and with approval of the local ethics committees. Following renal biopsy, the tissue was transferred to RNase inhibitor and microdissected into glomerular and tubular fragments. Total RNA was isolated from both micro-dissected compartments, linearly amplified and hybridized to Affymetrix HG-U133 Plus 2.0 microarrays as reported previously (25). Fragmentation, hybridization, staining, and imaging were performed according to the Affymetrix Expression Analysis Technical Manual (Affymetrix, Santa Clara, CA). The raw data was normalized using Robust Multichip Algorithm (RMA) and annotated by Human Entrez Gene custom CDF annotation version 18 (http://brainarray.mbni.med.umich.edu/Brainarray/Database/CustomCDF/genomic_curated_CDF.asp). To identify differentially expressed genes the SAM (Significance analysis of Microarrays) method was applied using TiGR (MeV, Version 4.8.1) (26). A *q*-value below 5% was considered to be statistically significant.

### Human Kidney Biopsy Immunohistochemistry

Human renal tissue, fixed in formaldehyde, and embedded in paraffin, was selected from the files of the Service of Pathology, University Hospital Geneva: control normal renal tissue was obtained from a patient with nephrectomy performed for neoplasia, involving the possibility of tumor-related immune exhaustion. Eight biopsy specimens were obtained from patients with diabetes (2 females, 6 males; mean age: 53 year-old; 2 diabetes type I and 6 diabetes type 2). These 8 biopsies, performed for proteinuria, demonstrated diabetic nephropathy with different degrees of interstitial fibrosis and tubular atrophy (IFTA): 4 biopsy specimens with IFTA <40% and 4 biopsy specimens with IFTA >50%. For all biopsy specimens, standard analyses were performed. Each patient gave informed consent before enrollment. The institutional ethical committee board approved the clinical protocol (CEREH number 03-081). The research was performed according to the Helsinki's declaration principles. Immunohistochemistry: after antigen heat retrieval, 3 μm serial sections of the formaldehyde-fixed, paraffin-embedded biopsy specimens were incubated with four different antibodies: rabbit anti-human NLRP3 (ABF23, Merck, Darmstadt, Germany) at a 1:1,500 dilution 1 h at room temperature, mouse anti-human CD68 (clone PG-M1, code M 0876, DakoCytomation, Glostrup, Denmark) at a 1:100 dilution, mouse anti-human IL1-alpha (LS-B1581, LifeSpan BioSciences, Seattle, Washington, USA) at a 1:500 dilution, and rabbit anti-human IL1-beta (ab82558, Abcam, Cambridge, UK) at a 1:400 dilution. Serial sections were incubated with the adequate antibody for 1 h at room temperature followed by the appropriate second antibody for 30 min and then by liquid diaminobenzidine substrate-chromogen system (DakoCytomation). Counterstaining was performed using Mayer hematoxylin. Negative controls included the absence of the primary antibody (not shown).

### Animal Studies

Eight-week-old male BKS *db/db* and nondiabetic BKS wild type mice (Taconic, Ry, Denmark) were housed in groups of 2–3 under standard conditions including enrichment. Mice underwent morning uninephrectomy (DM-1K for diabetic mice; WT-1K for nondiabetic mice) or sham surgery (DM-2K for diabetic mice, WT-2K for nondiabetic mice) with rigorous core body temperature control (27, 28). Group size calculation was based on glomerular filtration rate (GFR) as a primary endpoint and quantitative assumptions obtained from our previous studies (20, 21, 27). The group size for WT-2K, WT-1K, DM-2K, DM-1K+IgG, and DM-1K+antiIL-1β was, 5, 5, 9, 8, and 9, respectively. At age 18 weeks, only DM-1K mice with proteinuria at baseline were assigned by stratified randomization to different groups injected with either anti-IL-1β IgG (RO7114667, developed and provided by Hoffmann La Roche, Basel, Switzerland) or control IgG (10 mg/kg body weight weekly intraperitoneally for 8 weeks). The antibody was raised as a monoclonal antibody in a mouse hybridoma and then reformatted using VHVL sequences and a murine IgG1 scaffold with effector silencing DAPG muations (29). Antibody specificity was raised against human IL-1β but showed strong cross reactivity to murine Il-1β, while it did not bind recombinant human and mouse IL-1α (29). The antibody is neutralizing the biological effects of human and murine IL-1β as verified by ELISA-based protein:protein interaction inhibizion assays (29). Animal welfare was monitored throughout the study. All experiments were conducted according to the European equivalent of the NIH's *Guide for the Care and Use of Laboratory Animals* and had been approved by the local government authorities.

### Primary Endpoint

GFR was determined in conscious mice by transcutaneous measurement with FITC-sinistrin (Mannheim Pharma and Diagnostics) injection as first described (30) with slight inhouse modifications (28, 31). GFR (μl/min) was calculated from the decrease in fluorescence intensity over using a one-compartment model (32). Body weight of the mouse was used as an empirical conversion factor.

### Secondary Endpoints

Proteinuria was a secondary endpoint. Urine was collected at different time intervals, analyzed for albumin by ELISA (Bethyl Labs, Montgomery, TX, USA) and urinary creatinine (Jaffe' reaction; DiaSys Diagnostic Systems, Holzheim, Germany), and reported as albumin/creatinine ratio. Blood samples were obtained after 4 h fasting and analyzed for blood glucose level (Glucose GOD FS, DiaSys Diagnostic Systems, Holzheim, Germany).

Histology was a secondary endpoint. Kidneys were fixed in 4% formalin, embedded in paraffin, and stained with periodic acid–Schiff (PAS) reagent. Glomerular tuft area and capsule area were quantified on at least 25 glomeruli in PAS staining using ImageJ software. Sirius red staining was performed by staining paraffin sections with 0.1% of picro-sirius red solution (Direct Red 80, Sigma-Aldrich). Immunostaining was performed as described using anti-mouse Wilms Tumor (WT)-1 (immunohistochemistry staining; 1:200; Santa Cruz Biotechnology, Santa Cruz, CA), rabbit anti-mouse alpha smooth muscle actin (αSMA) (Dako GmbH, Germany), and rat anti-mouse Mac2 (1:5,000; Cederlane) (33). For WT-1 and Mac-2 staining, cells were counted in a minimum of 15 glomeruli per section by a blinded observer.

Gene transcription levels was a secondary endpoint. Total mRNA from whole kidney was transcribed into cDNA using Superscript II and subjected to real-time PCR on a Light Cycler 480 (Roche, Mannheim, Germany, Real Time PCR Detection Systems) using SYBR green (SABiosciences) as described (34). Primers used for the genes were listed in Table 1.

**Table 1:** Primer used in animal study.

**Gene**	**Forward**	**Reverse**
IL-1a	AGGGAGTCAACTCATTGGCG	ACTGTAGTCTTCGTTTTCACTGT
IL-1b	TGCCACCTTTTGACAGTGATG	AAGGTCCACGGGAAAGACAC
IL-1R1	CTGTTGGTGAGGAATGTGGCTG	GGCTCAGGATAACAGGTCTGTC
IL-1R2	CAGTGCAGCAAGACTCTGGTAC	GCAAGTAGGAGACATGAGGCAG
NLRP3	ACGTGTCATTCCACTCTGGC	AGGGAGTCAACTCATTGGCG
WT-1	CTGTACTGGGCACCACAGAG	CCAGCTCAGTGAAATGGACA
Synatopodin	AGGAGCCCAGGCCTTCTCT	GCCAGGGACCAGCCAGATA
IL-6	TGCCACCTTTTGACAGTGATG	AAGGTCCACGGGAAAGACAC
TNF-alpha	CTCTTCTGCCTGCTGCACTTTG	ATGGGCTACAGGCTTGTCACTC
TGF-beta	TGATACGCCTGAGTGGCTGTCT	CACAAGAGCAGTGAGCGCTGAA
CCR5	GTCTACTTTCTCTTCTGGACTCC	CCAAGAGTCTCTGTTGCCTGCA
CCL5	CCTGCTGCTTTGCCTACCTCTC	ACACACTTGGCGGTTCCTTCGA
VCAM-1	GCTATGAGGATGGAAGACTCTGG	ACTTGTGCAGCCACCTGAGATC
ICAM-1	AAACCAGACCCTGGAACTGCAC	GCCTGGCATTTCAGAGTCTGCT
KIM-1	TGGTTGCCTTCCGTGTCTCT	TCAGCTCGGGAATGCACAA
Ngal	ATGTCACCTCCATCCTGG	GCCACTTGCACATTGTAG
Col1alpha1	ACATGTTCAGCTTTGTGGAC	TAGGCCATTGTGTATGCAG
Alpha-SMA	GCTGTTGTAGGTGGTCTCAT	ACCATCGGCAATGAGCGTTT
18s	GCAATTATTCCCCATGAACG	AGGGCCTCACTAAACCATCC

### Statistical Analysis

Data are presented as mean ± SEM. Comparison between DM1K control IgG and anti-IL-1β treatment was performed with Student's *t*-test or Mann–Whitney *U*-test. Comparison of multiple groups was performed using ANOVA or Kruskal–Wallis test and *post-hoc* Dunnett's test or Dunn's test was used for multiple comparisons. A value of *p* < 0.05 was considered to indicate statistical significance. Data was presented as means ± SEM.

## Results

### IL-1β Expression in Diabetic Kidney Disease

**Figure 1:**
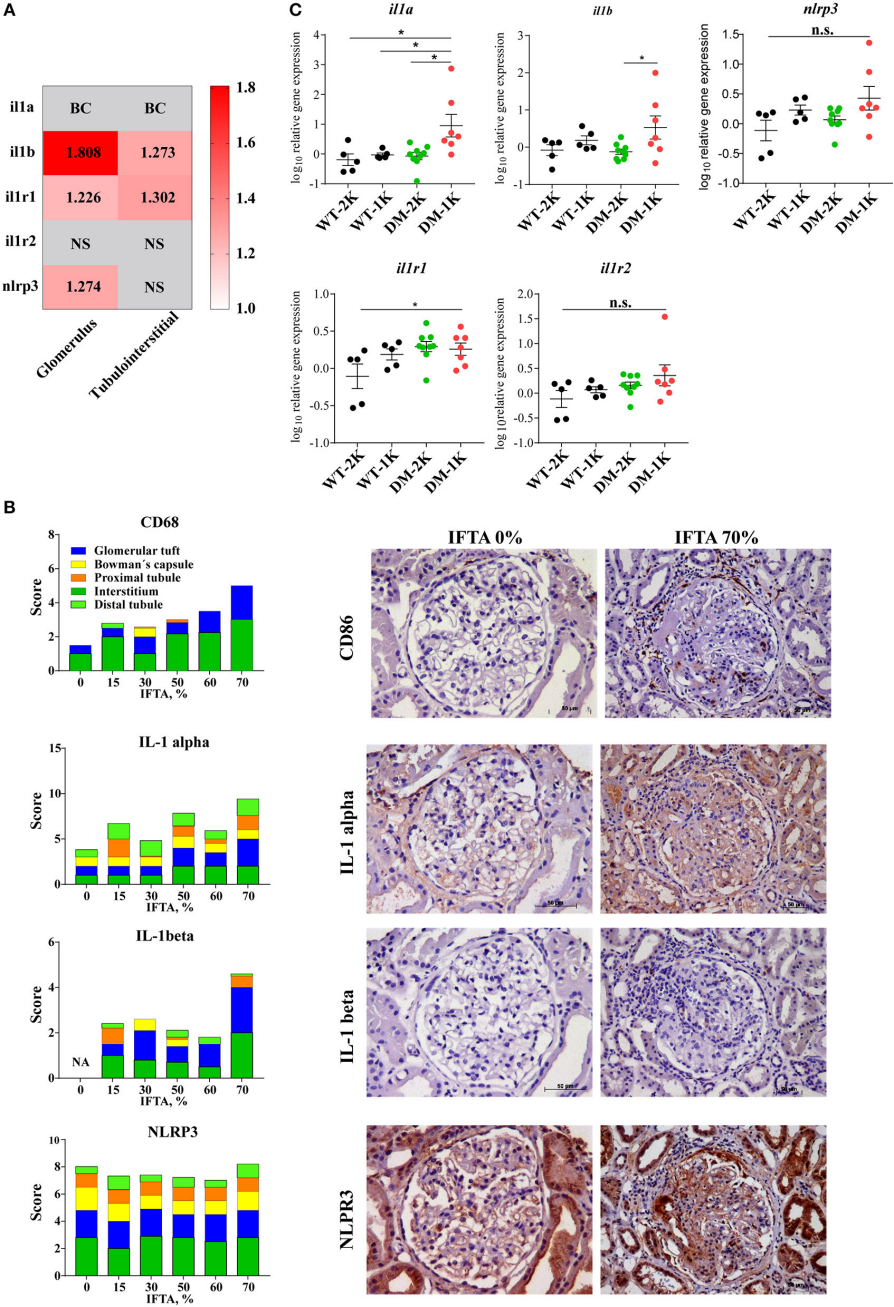
IL-1β expression in diabetic kidney disease. **(A)** Gene expression data of microdissected glomeruli and tubulointerstitium from kidney biopsies of patients with diabetic glomerulopathies and living donors as controls. IL-1β, IL1R1 gene expression was upregulated in both glomeruli and tubulointerstitium in diabetic nephropathy; NLRP3 gene expression was also increased in glomeruli in diabetic nephropathy. **(B)** Archived kidney biopsies were stained for CD68, IL-1α, IL-1β, and NLRP3. A semi-quantatitive score for staining positivity was employed and is illustrated for different kidney compartments in cases with IFTA 0% to IFTA 70%. Representative images are shown at an original magnification of 250x. **(C)** Kidney mRNA expression levels of IL-1β and related genes from 26 weeks old non-diabetic wildtype and diabetic *db/db* mice. Data in C are means ± SEM of 5–9 mice in each group and the values given are normalized to 18S rRNA and WT-2K group. *p < 0.05.

The paradigmatic proinflammatory cytokine IL-1β is not expressed under basal conditions and is induced only upon specific stimulation (4, 6). As such the human protein atlas does not display constitutive IL-1β positivity in healthy human kidney tissue, neither in the glomerular nor in the tubulointerstitial compartment (www.proteinatlas.com). In contrast, glomeruli and tubulointerstitial samples microdissected from diagnostic biopsies of diabetic patients with DKD revealed a significant induction of IL-1β transcripts in both compartments (Figure 1A). To localize IL-1β protein expression we performed immunostaining on several such diagnostic biopsies from patients with different stages of diabetes-related CKD and found IL-1β positivity exclusively localized to few infiltrating mononuclear cells, most likely macrophages, inside glomeruli as well as the tubulointerstitium and in some tubular cells (Figure 1B). IL-1β positivity increased with the stage of CKD as indicated by increasing amounts of interstitial fibrosis and tubular atrophy (Figure 1B). Consistent with this finding, we analyzed proIL-1β transcript levels in a mouse model of T2DM and found increased levels of IL-1β only in mice in which the progression of kidney disease had been accelerated by uninephrectomy (Figure 1C). Thus, IL-1β protein expression in the kidney predominately originates from infiltrating immune cells inside glomeruli and outside nephrons in the interstitium, as well as tubular cells.

### Therapeutic IL-1β Inhibition Does not Affect Type 2 Diabetes in *db/db* Mice

**Figure 2:**
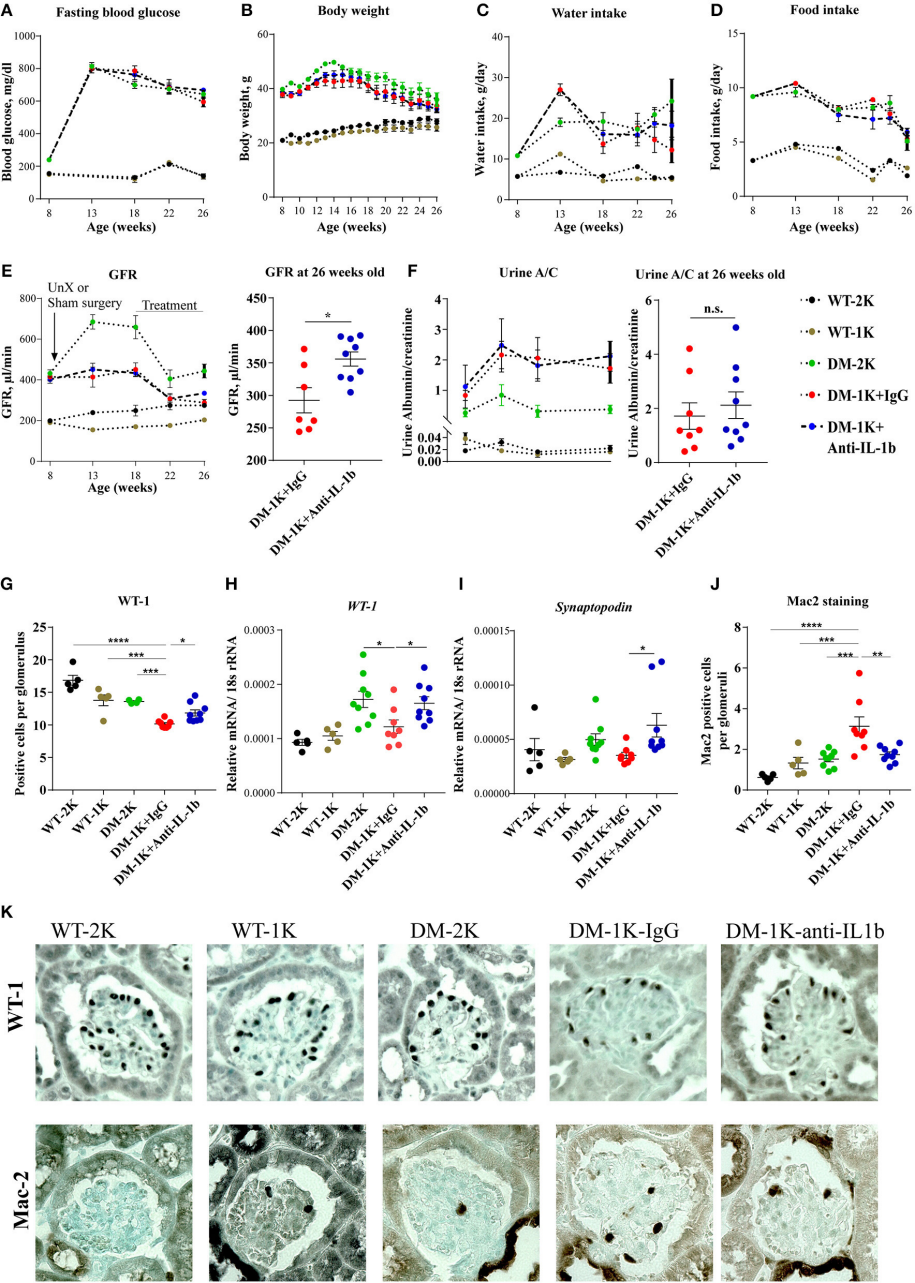
Effects of anti-IL-1β IgG on type 2 diabetic db/db mice. DM-1K mice were injected from week 18 to 26 of age with anti-IL-1β antibody, which did not affect fasting blood glucose **(A)**, body weight **(B)**, water intake **(C)**, or food intake **(D)** as compared to DM-1K with control IgG injection group. GFR **(E)** was measured at different time intervals. Note that at 26 weeks of age anti-IL-1β treatment significantly preserved higher GFR in DM-1K mice compared to control IgG. Urinary albumin/creatinine (A/C) ratio **(F)** was determined at different time intervals. **(G)** Kidney sections of 26 weeks old mice of all groups were stained for WT-1 to quantify podocytes per glomerular cross section. Graphs showed the mean numbers of WT-1 positive cells in 15-25 glomeruli ± SEM in sections. **(H–I)** Kidney mRNA expression levels were quantified by real–time RT-PCR. **(J)** Kidney sections of 26 weeks old mice of all groups were stained for Mac-2 to quantify macrophages per glomerular cross section. The graphs shows the mean numbers of Mac-2 positive cells in 15–25 glomeruli ± SEM in sections. **(K)** Representative images of WT-1 staining and Mac-2 staining. *N* = 5 in WT-2K and WT-1K groups, *n* = 7–9 in DM-2K group, *n* = 8 in DM-1K+IgG group, and *n* = 9 in DM-1K+antiIL-1β group. Data represent means ± SEM. *p < 0.05, **p < 0.01, ***p < 0.001, ****p < 0.0001.

To test the functional contribution of IL-1β in diabetes we started to inject male *db/db* mice starting 10 weeks after uninephrectomy (18 weeks of age) with anti-IL-1β IgG or control IgG and analyzed its impact on phenotypic parameters of T2DM. Anti-IL-1β IgG did not affect body weight, food and water intake or blood glucose levels over time (Figures 2A–D), implying that IL-1β has no major role in these metabolic aspects.

### Therapeutic IL-1β Inhibition Preserves Renal Structure and Function in *db/db* Mice

GFR slope is the main determinant of CKD progression to end stage kidney disease, also in diabetes. Anti-IL-1β IgG significantly preserved GFR by around 50 μl/min although not affecting urine albumin excretion (Figures 2E,F). This GFR was still in a range of increased total GFR due to diabetes-related single nephron hyperfiltration. IL-1β blockade also preserved more podocytes as indicated by either WT-1 staining or WT-1 and synaptopodin mRNA expression (Figures 2G–I), however, it did not alter glomerular tuft area, Bowman's capsule area, or tuft over capsule ratio, three indirect markers of diabetes- and nephron loss-related single nephron hyperfiltration (Supplementary Figure 1A). Anti-IL-1β IgG also decreased the number of infiltrated macrophages in glomeruli as evident by Mac-2 staing (Figures 2J,K), however it did not significantly affect proinflammatory cytokine expression, such as *IL-6, TNF*α, or *TGF*β, neither chemokine expression, such as *CCR5, CCL5, CXCR1*, or *VCAM-1* (Supplementary Figures 2A–G). For tubule injury-related genes, Ngal and Kim-1 expression levels were reduced in the anti-IL-1β IgG-treated group but only Ngal reached a significant difference (Supplementary Figures 2H,I). Thus, IL-1β blockade attenuated numerous markers of progressive CKD in *db/db* mice. IL-1β blockade did not significantly affect interstitial fibrosis as detected by aSMA staining or mesangial sclerosis as detected by Sirius red staining (Supplementary Figures 1B-E), though anti-IL-1β treatment reduced α*SMA* but not collagen 1alpha1 gene expression (Supplementary Figures 2J,K). Thus, IL-1β blockade attenuated numerous markers of progressive CKD in *db/db* mice.

### Adverse Events

No adverse events were observed during the study period as per regular scoring.

## Discussion

We had hypothesized that IL-1β would contribute to the progression of CKD in T2DM and used a novel IL-1β-specific antibody that can neutralize its biological effects in a validated mouse model of CKD in T2DM. We found renoprotective effects on GFR, podocyte loss, and intrarenal inflammation.

Several studies reported IL-1 blockade to improve β-cell function and glucose control in experimental or human T1 or T2DM (35–38). In contrast, in obese *db/db* mice with T2DM injection of recombinant IL-1β can cause long-lasting hypoglycemia in *db/db* mice (39). In our experimental setting, IL-1 blockade did not affect glucose control, which may relate to a different neurohumoral regulation of glucose control in *db/db* mice compared to humans (39).

CKD progression in diabetes results from direct glycotoxicity on renal cells as well as from hyperglycemia-induced and SGLT2-mediated glomerular hyperfiltration and tubular hyperreabsorbtion (1, 40, 41). A low nephron number, as induced here by early uninephrectomy, further aggravates hyperfunction, and accelerates the demise of the remnant nephrons (42). Several studies have documented an increased expression of NLRP3 inflammasome components in renal parenchymal cells in experimental and human DKD and hence speculated on a role of IL-1β in CKD progression (18, 43–46). Indeed, Shahzad et al. found *db/db* mice with T2DM to be protected from kidney disease by injecting human recombinant IL-1R antagonist anakinra (18). Anakinra blocks signaling of IL-1R, a receptor for both IL-1α and IL-1β (47). As we could not find a similar protective effect by specifically neutralizing IL-1β in the same disease model, we conclude that the effects on CKD observed with anakinra must have been IL-1β-independent and involve e.g., IL-1α.

We conclude, specific targeting of IL-1β has a moderate effect on GFR decline, podocyte loss, and renal inflammation in T2DM mice with CKD. Whether these findings can translate into better outcomes also in human DKD remains to be determined.

## Ethics Statement

Animal studies were approved by the Regierung von Oberbayern. Human studies where approved by the institutional ethical committee board University of Geneva (CEREH number 03-081) and the local ethics committees of all ERCB participating centers.

## Author Contributions

ML and CC provided transcriptional analysis of human biopsies. SM performed immunostaining of human kidney biopsies. YL, SD, SK, and MM performed the mouse studies. SK and H-JA designed the study. YL and H-JA wrote the manuscript. All authors revised and approved the final version of the manuscript.

### Conflict of Interest Statement

H-JA received consultancy fees and SK received research support from Hoffmann La Roche. The remaining authors declare that the research was conducted in the absence of any commercial or financial relationships that could be construed as a potential conflict of interest.

## Supplementary Material

Supplementary Figure 1 Kidney pathology evaluation of anti-IL-1β IgG on type 2 diabetic db/db mice. **(A)** Area of glomerular tuft (encircled by yellow lines), area of Bowman's capsule (encircled by green line), and the ratio of tuft over capsule were measured in PAS staining at an original magnification of 400X. **(B)** Kidney sections of all groups were stained for alpha-smooth muscle actin (aSMA) to quantify kidney fibrosis. Original magnification 25x. **(C)** Kidney sections of all groups were stained for Sirius red to quantify the glomerular collagen deposition. **(D,E)** Glomerulosclerosis was quantified in all groups upon staining kidney sections with sirius red by using automated digital morphometry upon selecting only tuft areas. Original magnification 400x. Data represent means ± SEM. ^*^*p* < 0.05, ^***^*p* < 0.001, ^****^*p* < 0.0001.

Supplementary Figure 2 Kidney gene expression by RT-PCR in animal study. **(A–G)** Pro-inflammatory cytokine and chemokine gene expression was quantified. **(H,I)** Tubular injury genes of *KIM-1* and *Ngal* were measured. **(J,K)** Extracellular matrix gene expression were quantified as described in methods.

## References

[1] Anders HJ Huber TB Isermann BM Schiffer M. CKD in diabetes: diabetic kidney disease versus nondiabetic kidney disease. Nat Rev Nephrol. (2018) 14:361–77. 10.1038/s41581-018-0001-y 29654297

[2] Binder CJ Papac-Milicevic N Witztum JL. Innate sensing of oxidation-specific epitopes in health and disease. Nat Rev Immunol. (2016) 16:485–97. 10.1038/nri.2016.63 27346802 PMC7097710

[3] Sharma A Tate M Mathew G Vince JE Ritchie RH de Haan JB. Oxidative stress and NLRP3-inflammasome activity as significant drivers of diabetic cardiovascular complications: therapeutic implications. Front Physiol. (2018) 9:114. 10.3389/fphys.2018.00114 29515457 PMC5826188

[4] Anders HJ. Of inflammasomes and alarmins: IL-1beta and IL-1alpha in kidney disease. J Am Soc Nephrol. (2016) 27:2564–75. 10.1681/ASN.2016020177 27516236 PMC5004665

[5] Leemans JC Kors L Anders HJ Florquin S. Pattern recognition receptors and the inflammasome in kidney disease. Nat Rev Nephrol. (2014) 10:398–414. 10.1038/nrneph.2014.91 24890433

[6] Dinarello CA Wolff SM. The role of interleukin-1 in disease. N Engl J Med. (1993) 328:106–13. 10.1056/NEJM199301143280207 8439348

[7] Lachmann HJ Kone-Paut I Kuemmerle-Deschner JB Leslie KS Hachulla E Quartier P. Use of canakinumab in the cryopyrin-associated periodic syndrome. N Engl J Med. (2009) 360:2416–25. 10.1056/NEJMoa0810787 19494217

[8] Lequerre T Quartier P Rosellini D Alaoui F De Bandt M Mejjad O. Interleukin-1 receptor antagonist (anakinra) treatment in patients with systemic-onset juvenile idiopathic arthritis or adult onset Still disease: preliminary experience in France. Ann Rheum Dis. (2008) 67:302–8. 10.1136/ard.2007.076034 17947302

[9] Lovell DJ Giannini EH Reiff AO Kimura Y Li S Hashkes PJ. Long-term safety and efficacy of rilonacept in patients with systemic juvenile idiopathic arthritis. Arthritis Rheum. (2013) 65:2486–96. 10.1002/art.38042 23754188

[10] Ridker PM Everett BM Thuren T MacFadyen JG Chang WH Ballantyne C. Antiinflammatory therapy with canakinumab for atherosclerotic disease. N Engl J Med. (2017) 377:1119–31. 10.1056/NEJMoa1707914 28845751

[11] Ruperto N Brunner HI Quartier P Constantin T Wulffraat N Horneff G. Two randomized trials of canakinumab in systemic juvenile idiopathic arthritis. N Engl J Med. (2012) 367:2396–406. 10.1056/NEJMoa1205099 23252526

[12] Schlesinger N Alten RE Bardin T Schumacher HR Bloch M Gimona A Canakinumab for acute gouty arthritis in patients with limited treatment options: results from two randomized, multicentre, active-controlled, double-blind trials and their initial extensions. Ann Rheum Dis. (2012) 71:1839–48. 10.1136/annrheumdis-2011-200908 22586173

[13] Cabrera SM Wang X Chen YG Jia S Kaldunski ML Greenbaum CJ. Interleukin-1 antagonism moderates the inflammatory state associated with Type 1 diabetes during clinical trials conducted at disease onset. Eur J Immunol. (2016) 46:1030–46. 10.1002/eji.201546005 26692253 PMC4828314

[14] Choudhury RP Birks JS Mani V Biasiolli L Robson MD L'Allier PL Arterial effects of canakinumab in patients with atherosclerosis and type 2 diabetes or glucose intolerance. J Am Coll Cardiol. (2016) 68:1769–80. 10.1016/j.jacc.2016.07.768 27737744 PMC5064025

[15] Larsen CM Faulenbach M Vaag A Volund A Ehses JA Seifert B. Interleukin-1-receptor antagonist in type 2 diabetes mellitus. N Engl J Med. (2007) 356:1517–26. 10.1056/NEJMoa065213 17429083

[16] Moran A Bundy B Becker DJ DiMeglio LA Gitelman SE Goland R. Interleukin-1 antagonism in type 1 diabetes of recent onset: two multicentre, randomised, double-blind, placebo-controlled trials. Lancet. (2013) 381:1905–15. 10.1016/S0140-6736(13)60023-9 23562090 PMC3827771

[17] Ridker PM Howard CP Walter V Everett B Libby P Hensen J. Effects of interleukin-1beta inhibition with canakinumab on hemoglobin A1c, lipids, C-reactive protein, interleukin-6, and fibrinogen: a phase IIb randomized, placebo-controlled trial. Circulation. (2012) 126:2739–48. 10.1161/CIRCULATIONAHA.112.122556 23129601

[18] Shahzad K Bock F Dong W Wang H Kopf S Kohli S. Nlrp3-inflammasome activation in non-myeloid-derived cells aggravates diabetic nephropathy. Kidney Int. (2015) 87:74–84. 10.1038/ki.2014.271 25075770 PMC4284813

[19] Orellana JM Kampe K Schulze F Sieber J Jehle AW. Fetuin-A aggravates lipotoxicity in podocytes via interleukin-1 signaling. Physiol Rep. (2017) 5:e13287. 10.14814/phy2.13287 28554965 PMC5449566

[20] Darisipudi MN Kulkarni OP Sayyed SG Ryu M Migliorini A Sagrinati C. Dual blockade of the homeostatic chemokine CXCL12 and the proinflammatory chemokine CCL2 has additive protective effects on diabetic kidney disease. Am J Pathol. (2011) 179:116–24. 10.1016/j.ajpath.2011.03.004 21703397 PMC3123871

[21] Ninichuk V Clauss S Kulkarni O Schmid H Segerer S Radomska E. Late onset of Ccl2 blockade with the Spiegelmer mNOX-E36-3'PEG prevents glomerulosclerosis and improves glomerular filtration rate in db/db mice. Am J Pathol. (2008) 172:628–37. 10.2353/ajpath.2008.070601 18258851 PMC2258250

[22] de Zeeuw D Bekker P Henkel E Hasslacher C Gouni-Berthold I Mehling H. The effect of CCR2 inhibitor CCX140-B on residual albuminuria in patients with type 2 diabetes and nephropathy: a randomised trial. Lancet Diabetes Endocrinol. (2015) 3:687–96. 10.1016/S2213-8587(15)00261-2 26268910

[23] Menne J Eulberg D Beyer D Baumann M Saudek F Valkusz Z. C-C motif-ligand 2 inhibition with emapticap pegol (NOX-E36) in type 2 diabetic patients with albuminuria. Nephrol Dial Transplant. (2017) 32:307–15. 10.1093/ndt/gfv459 28186566 PMC5410979

[24] Cohen CD Frach K Schlondorff D Kretzler M. Quantitative gene expression analysis in renal biopsies: a novel protocol for a high-throughput multicenter application. Kidney Int. (2002) 61:133–40. 10.1046/j.1523-1755.2002.00113.x 11786093

[25] Cohen CD Klingenhoff A Boucherot A Nitsche A Henger A Brunner B. Comparative promoter analysis allows *de novo* identification of specialized cell junction-associated proteins. Proc Natl Acad Sci USA. (2006) 103:5682–7. 10.1073/pnas.0511257103 16581909 PMC1421338

[26] Tusher VG Tibshirani R Chu G. Significance analysis of microarrays applied to the ionizing radiation response. Proc Natl Acad Sci USA. (2001) 98:5116–21. 10.1073/pnas.091062498 11309499 PMC33173

[27] Sayyed SG Gaikwad AB Lichtnekert J Kulkarni O Eulberg D Klussmann S. Progressive glomerulosclerosis in type 2 diabetes is associated with renal histone H3K9 and H3K23 acetylation, H3K4 dimethylation and phosphorylation at serine 10. Nephrol Dial Transplant. (2010) 25:1811–7. 10.1093/ndt/gfp730 20067909

[28] Marschner JA Schafer H Holderied A Anders HJ. Optimizing mouse surgery with online rectal temperature monitoring and preoperative heat supply. Effects on post-ischemic acute kidney injury. PLoS ONE. (2016) 11:e0149489. 10.1371/journal.pone.0149489 26890071 PMC4758659

[29] Dengl S Huelsmann PM Gassner C Mundogl O Georges G Schumacher R Anti-IL-lbeta antibodies and methods of use. WIPO, WO 2016/075034 Al, 1-109. (2016).

[30] Schreiber A Shulhevich Y Geraci S Hesser J Stsepankou D Neudecker S. Transcutaneous measurement of renal function in conscious mice. Am J Physiol Renal Physiol. (2012) 303:F783–8. 10.1152/ajprenal.00279.2012 22696603

[31] Steiger S Grill JF Ma Q Bauerle T Jordan J Smolle M. Anti-transforming growth factor beta IgG elicits a dual effect on calcium oxalate crystallization and progressive nephrocalcinosis-related chronic kidney disease. Front Immunol. (2018) 9:619. 10.3389/fimmu.2018.00619 29651290 PMC5884871

[32] Friedemann J Heinrich R Shulhevich Y Raedle M William-Olsson L Pill J Schock-Kusch D. Improved kinetic model for the transcutaneous measurement of glomerular filtration rate in experimental animals. Kidney Int. (2016) 90:1377–85. 10.1016/j.kint.2016.07.024 27665115

[33] Kumar S Vr Darisipudi MN Steiger S Devarapu SK Tato M Kukarni OP Cathepsin S Cleavage of protease-activated receptor-2 on endothelial cells promotes microvascular diabetes complications. J Am Soc Nephrol. (2016) 27:1635–49. 10.1681/ASN.2015020208 26567242 PMC4884104

[34] Lech M Avila-Ferrufino A Skuginna V Susanti HE Anders HJ. Quantitative expression of RIG-like helicase, NOD-like receptor and inflammasome-related mRNAs in humans and mice. Int Immunol. (2010) 22:717–28. 10.1093/intimm/dxq058 20584763

[35] Hensen J Howard CP Walter V Thuren T. Impact of interleukin-1beta antibody (canakinumab) on glycaemic indicators in patients with type 2 diabetes mellitus: results of secondary endpoints from a randomized, placebo-controlled trial. Diabetes Metab. (2013) 39:524–31. 10.1016/j.diabet.2013.07.003 24075453

[36] Owyang AM Maedler K Gross L Yin J Esposito L Shu L. XOMA 052, an anti-IL-1{beta} monoclonal antibody, improves glucose control and {beta}-cell function in the diet-induced obesity mouse model. Endocrinology. (2010) 151:2515–27. 10.1210/en.2009-1124 20332197

[37] Zhang Y Yu XL Zha J Mao LZ Chai JQ Liu RT. Therapeutic vaccine against IL-1beta improved glucose control in a mouse model of type 2 diabetes. Life Sci. (2018) 192:68–74. 10.1016/j.lfs.2017.11.021 29155303

[38] Cavelti-Weder C Babians-Brunner A Keller C Stahel MA Kurz-Levin M Zayed H. Effects of gevokizumab on glycemia and inflammatory markers in type 2 diabetes. Diabetes Care. (2012) 35:1654–62. 10.2337/dc11-2219 22699287 PMC3402269

[39] Besedovsky HO Del Rey A. Interleukin-1 resets glucose homeostasis at central and peripheral levels: relevance for immunoregulation. Neuroimmunomodulation. (2010) 17:139–41. 10.1159/000258707 20134186

[40] Heerspink HJL Kosiborod M Inzucchi SE Cherney DZI. Renoprotective effects of sodium-glucose cotransporter-2 inhibitors. Kidney Int. (2018) 94:26–39. 10.1016/j.kint.2017.12.027 29735306

[41] Anders HJ Davis JM Thurau K. Nephron protection in diabetic kidney disease. N Engl J Med. (2016) 375:2096–8. 10.1056/NEJMcibr1608564 27959742

[42] Anguiano Gomez L Lei Y Kumar Devarapu S Anders HJ The diabetes pandemic suggests unmet needs for “CKD with diabetes” in addition to “diabetic nephropathy”-implications for pre-clinical research and drug testing. Nephrol Dial Transplant. (2018) 33:1292–304. 10.1093/ndt/gfx219 28992221

[43] El-Horany HE Abd-Ellatif RN Watany M Hafez YM Okda HI. NLRP3 expression and urinary HSP72 in relation to biomarkers of inflammation and oxidative stress in diabetic nephropathy patients. IUBMB Life. (2017) 69:623–30. 10.1002/iub.1645 28631886

[44] Qiu YY Tang LQ. Roles of the NLRP3 inflammasome in the pathogenesis of diabetic nephropathy. Pharmacol Res. (2016) 114:251–64. 10.1016/j.phrs.2016.11.004 27826011

[45] Wang Y Yu B Wang L Yang M Xia Z Wei W. Pioglitazone ameliorates glomerular NLRP3 inflammasome activation in apolipoprotein E knockout mice with diabetes mellitus. PLoS ONE.(2017) 12:e0181248. 10.1371/journal.pone.0181248 28708885 PMC5510862

[46] Wu M Han W Song S Du Y Liu C Chen N. NLRP3 deficiency ameliorates renal inflammation and fibrosis in diabetic mice. Mol Cell Endocrinol. (2018) 478:115–25. 10.1016/j.mce.2018.08.002 30098377

[47] Dinarello CA. The role of the interleukin-1-receptor antagonist in blocking inflammation mediated by interleukin-1. N Engl J Med. (2000) 343:732–4. 10.1056/NEJM200009073431011 10974140

[48] Shved N Warsow G Eichinger F Hoogewijs D Brandt S Wild P. Transcriptome-based network analysis reveals renal cell type-specific dysregulation of hypoxia-associated transcripts. Sci Rep. (2017) 7:8576. 10.1038/s41598-017-08492-y 28819298 PMC5561250

