# Supplementary figures and images for: Interleukin-1β Inhibition for Chronic Kidney Disease in Obese Mice With Type 2 Diabetes
Source: Front Immunol. 2019 May 29;10:1223. doi: 10.3389/fimmu.2019.01223 (PMC6549251; doi:10.3389/fimmu.2019.01223)

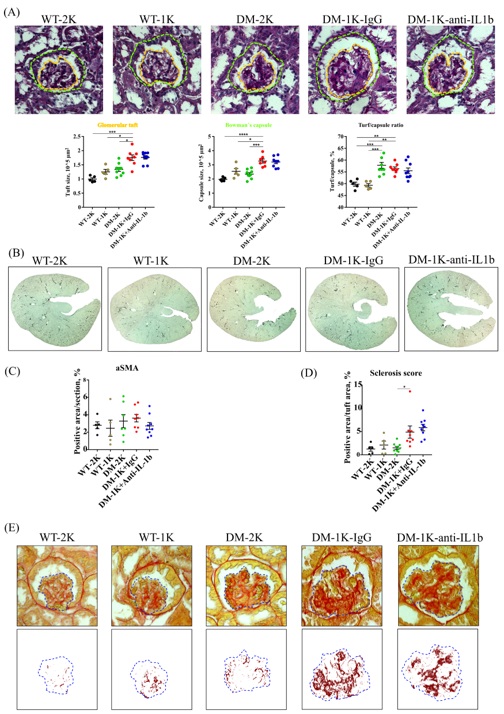

Supplement: Supplementary Figure 1 — Kidney pathology evaluation of anti-IL-1β IgG on type 2 diabetic db/db mice. (A) Area of glomerular tuft (encircled by yellow lines), area of Bowman's capsule (encircled by green line), and the ratio of tuft over capsule were measured in PAS staining at an original magnification of 400X. (B) Kidney sections of all groups were stained for alpha-smooth muscle actin (aSMA) to quantify kidney fibrosis. Original magnification 25x. (C) Kidney sections of all groups were stained for Sirius red to quantify the glomerular collagen deposition. (D,E) Glomerulosclerosis was quantified in all groups upon staining kidney sections with sirius red by using automated digital morphometry upon selecting only tuft areas. Original magnification 400x. Data represent means ± SEM. *p < 0.05, ***p < 0.001, ****p < 0.0001. [file Image_1.jpg]

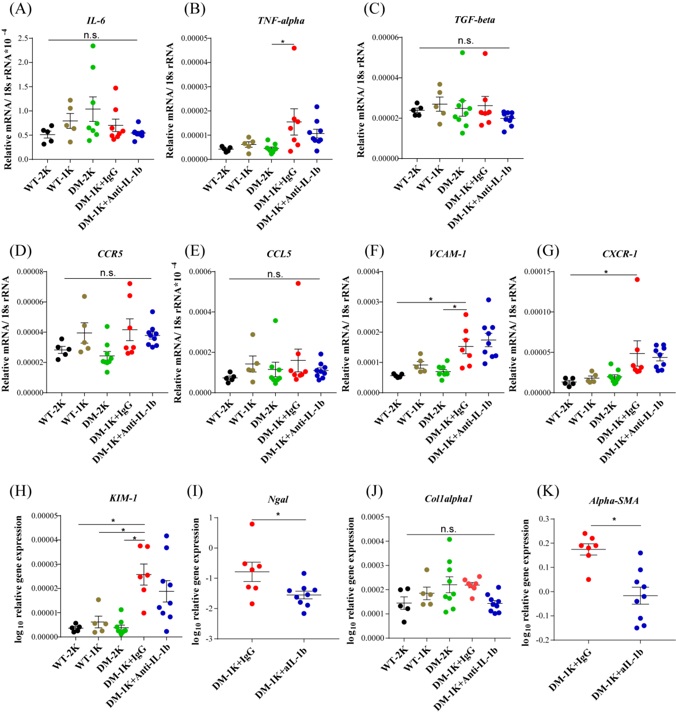

Supplement: Supplementary Figure 2 — Kidney gene expression by RT-PCR in animal study. (A–G) Pro-inflammatory cytokine and chemokine gene expression was quantified. (H,I) Tubular injury genes of KIM-1 and Ngal were measured. (J,K) Extracellular matrix gene expression were quantified as described in methods. [file Image_2.jpg]
